# Non-invasive biomarkers derived from the extracellular matrix associate with response to immune checkpoint blockade (anti-CTLA-4) in metastatic melanoma patients

**DOI:** 10.1186/s40425-018-0474-z

**Published:** 2018-12-19

**Authors:** Christina Jensen, Daniel Hargbøl Madsen, Morten Hansen, Henrik Schmidt, Inge Marie Svane, Morten Asser Karsdal, Nicholas Willumsen

**Affiliations:** 1 Biomarkers and Research, Nordic Bioscience, Herlev Hovedgade 205-207, 2730 Herlev, Denmark; 2 Biotech Research and Innovation Centre (BRIC), University of Copenhagen, Ole Maaløes Vej 5, 2200 Copenhagen N, Denmark; 3 Center for Cancer Immune Therapy, Department of Haematology and Department of Oncology, Herlev Hospital, University of Copenhagen, Herlev Ringvej 75, 2730 Herlev, Denmark; 4 Department of Oncology, Aarhus University Hospital, Nørrebrogade 44, 8000 Aarhus C, Denmark

**Keywords:** Immunotherapy, Immune checkpoint inhibitors, Liquid biopsy, Biomarkers, Extracellular matrix, Collagen, Stroma

## Abstract

**Background:**

Excessive extracellular matrix (ECM) remodeling and a reactive stroma can affect T-cell infiltration and T-cell activity in the tumor and hereby influence response to immune checkpoint inhibitors (ICI). In the pursuit of finding biomarkers that predict treatment response, we evaluated the association between serum biomarkers of collagen and vimentin turnover and outcomes in metastatic melanoma patients treated with the anti-CTLA-4 antibody ipilimumab (IPI).

**Methods:**

Type III collagen formation (PRO-C3), MMP-degraded type I, type III and type IV collagens (C1M, C3M and C4M), and citrullinated and MMP-degraded vimentin (VICM) were measured with ELISAs in serum from metastatic melanoma patients before (*n* = 66) and 3 weeks after (*n* = 52) initiation of IPI treatment. Biomarker levels were associated with Disease Control Rate (DCR) and survival outcomes.

**Results:**

We found that baseline levels of PRO-C3 (*p* = 0.011), C1M (*p* = 0.003), C3M (*p* = 0.013) and C4M (*p* = 0.027) were significantly elevated in patients with progressive disease (PD). Univariate Cox regression analysis identified high PRO-C3 (*p* = 0.021) and C4M (*p* = 0.008) as predictors of poor overall survival (OS) and the biomarkers remained significant when evaluated with other covariates (PRO-C3 (*p* = 0.049) and C4M (*p* = 0.046)). Multivariate analysis identified VICM as a predictor of longer OS (*p* = 0.026). Similarly, a high C3M/PRO-C3 ratio predicted for increased OS (*p* = 0.034). Only C3M (*p* = 0.003) and VICM (*p* < 0.0001) increased 3 weeks after treatment.

**Conclusions:**

ECM and tissue remodeling quantified in pre-treatment serum were associated with response and survival outcomes in metastatic melanoma patients treated with IPI. This highlights the importance of addressing the ECM and stromal component non-invasively in future ICI studies.

**Electronic supplementary material:**

The online version of this article (10.1186/s40425-018-0474-z) contains supplementary material, which is available to authorized users.

## Background

Immune checkpoint blockade with monoclonal antibodies against cytotoxic T lymphocyte antigen 4 (CTLA-4) and programmed cell death protein 1 (PD-1) or their ligands, such as PD1 ligand 1 (PD-L1) has revolutionized the treatment of metastatic melanoma patients with the possibility of durable and long-lasting responses [1]. However, only a proportion of patients benefit from immune checkpoint inhibitor (ICI) treatment [1, 2]. Given the frequency of adverse events, high costs and the lack of reliable biomarkers, novel predictive biomarkers of treatment efficacy represent an unmet need [3]. Identifying biomarkers associated with clinical response to ICIs is essential for selection of patients and for identifying potential targets for the development of combination therapies.

Emerging evidence suggests that the extracellular matrix (ECM), the non-cellular component of all tissues, and proteolytic ECM remodeling products have a crucial role in resistance to immunotherapy by regulating the cancer-immunity cycle [4]. ECM remodeling, collagen deposition and mechanical forces have been shown to regulate immune cell migration and activation [5]. Some patients have an immune-excluded phenotype, in which a dense stroma (desmoplasia) in the tumor microenvironment restricts the access of tumor infiltrating lymphocytes (TILs) into the tumor, resulting in poor clinical responses to ICIs [6–8]. Desmoplasia is characterized by excessive deposition of ECM, which are constantly undergoing a remodeling [9]. In healthy tissue, a balanced ratio between ECM degradation and formation maintains tissue function whereas altered ECM composition plays a vital role in cancer progression and invasion [10]. Melanoma is one of the most aggressive human cancers with a highly reactive stroma [11]. This results in increased cleavage of collagen proteins by matrix-remodeling enzymes (such as matrix metalloproteinases (MMPs)), and the products may act as chemokines, cytokines and immune regulating agents [12, 13]. These small protein fragments containing specific protease-generated neo-epitopes, or ‘protein fingerprints’, are released into the circulation where they can be used as serological biomarkers directly reflecting disease pathogenesis [14].

Specific protein fingerprint biomarkers reflect the formation or degradation of various ECM proteins such as collagens. Collagen is the major structural protein of the skin primarily composed of interstitial matrix collagens type I and III and the basement membrane collagen type IV [15, 16]. The PRO-C3 biomarker is a protein-fragment with a fingerprint of type III collagen formation which can be used to assess excessive collagen formation (desmoplasia) in a liquid biopsy [17]. Elevated PRO-C3 levels have been detected in metastatic colorectal cancer patients and furthermore been associated with shorter overall survival (OS) in metastatic breast cancer patients [18, 19]. Specific MMP-generated protein-fragments derived from type I, III and IV collagen can be measured with the protein fingerprint biomarkers C1M, C3M and C4M, respectively. They reflect degradation of the interstitial matrix (C1M and C3M) and basement membrane (C4M), and are elevated in patients with various tumors and have been linked to tumor activity (a reactive stroma) [18, 20].

Another interesting stromal biomarker is citrullinated and MMP-degraded vimentin (VICM) that is released from activated macrophages and has been found elevated in lung cancer [21, 22]. Macrophages are known to play a role in the tumor microenvironment and biomarkers measuring macrophage activity may be relevant for ICIs [23].

The hypothesis of the current study was that elevated levels of biomarkers originating from a dense and reactive stroma were associated with outcome in metastatic melanoma patients treated with ICIs. To our knowledge, this is the first study to examine the biomarker potential of quantifying altered collagen turnover in serum for predicting outcome to ICIs.

In this study, we evaluated PRO-C3, C1M, C3M, C4M and VICM in serum samples drawn prior to treatment and after 3 weeks of therapy, for associations with clinical response to ipilimumab (IPI) in patients with metastatic melanoma.

## Methods

### Patient samples

Serum samples were collected from 66 stage IV malignant melanoma patients treated with IPI as standard of care at Herlev Hospital (*n* = 32), and Aarhus University Hospital (*n* = 34), Denmark subsequent to informed consent. Patient inclusion and exclusion criteria are described elsewhere [24]. Patients were included between October 2012 and June 2014. The study was approved by the Ethics Committee for The Capital Region of Denmark (H-2-2012-058) in compliance with the Helsinki Declaration of 1975. Treatment with IPI was given as a total of four treatments with 3 weeks apart, at a fixed dose of 3 mg/kg body weight. All patients received at least two doses of IPI. Serum samples were collected at baseline (pre-treatment) and 3 weeks after the first treatment (before the 2nd dose of treatment). Sample analyses were performed blindly. Clinical response was evaluated according to Response Evaluation Criteria in Solid Tumors (RECIST) (v. One.1.1). Patients were assessed within 4 weeks before the first treatment and every third months thereafter until progression. The median follow-up period was 473 days (range, 40–1258 days), defined as days to death or to last follow-up.

### ELISA measurements and procedure

Each ECM fragment has a specific protease-generated neo-epitope that the monoclonal antibodies used in the enzyme-linked immunosorbent assays (ELISAs) are highly specific against. PRO-C3 is generated by the N-protease-mediated release of the N-terminal pro-peptide of type III collagen [17], C1M is from MMP-degraded type I collagen [25], C3M is from MMP-degraded type III collagen [26], C4M is from MMP-degraded type IV collagen [27] and VICM is from MMP-degraded citrullinated vimentin (VICM) [28]. Levels of these ECM biomarkers were assessed in serum samples using well characterized competitive ELISAs manufactured by Nordic Bioscience (Herlev, Denmark) and performed according to the manufacturer’s specifications. In brief, 96-well pre-coated streptavidin plates were coated with biotinylated peptides specific for the protein of interest and incubated for 30 min at 20 °C. A volume of 20 μl of standard peptide or pre-diluted serum sample were added followed by addition of peroxidase-conjugated monoclonal antibodies and incubated for 1 h at 20 °C (C3M and C4M) or overnight at 4 °C (PRO-C3, C1M and VICM). Next, tetramethylbenzinidine (TMB) (Kem-En-Tec Diagnostics, Denmark) was added and the plates were incubated for 15 min at 20 °C. All incubations included shaking of the plates at 300 rpm following by five times of wash (20 mM Tris, 50 mM NaCl, pH 7.2). The reaction of TMB was stopped by adding 1% sulfuric acid and the absorbance was measured at 450 nm with 650 nm as reference. A standard curve was plotted using a 4-parametric mathematical fit model and data were analyzed using the Softmax Pro v. 6.3 software. Levels of the biomarkers were measured in duplicates.

### Statistical analyses

Data did not meet assumptions for parametric testing (D’Agostino & Pearson normality test) and therefore non-parametric tests were used to assess differences. Wilcoxon matched-pairs signed rank test was used to compare patients at baseline with week 3. Mann-Whitney test was used to compare patients with progressive disease (PD) to the combined group of patients with stable disease (SD), partial response (PR) and complete response (CR) at baseline. The odds ratio (OR) and the positive predictive value (PPV) were generated from a specific cut-off value, the 75th percentile for PRO-C3, C1M, C3M and C4M, which was based on a previous study [19], and the median for VICM, and analyzed using Fisher’s exact test.

Univariate Cox proportional-hazards regression models were used to calculate hazard ratios (HR) with 95%CI for prediction of OS and progression free survival (PFS) for the PRO-C3, C1M, C3M, C4M and VICM biomarkers as well as other relevant clinical covariates: age, lactate dehydrogenase (LDH) and prior systemic therapy. To assess any confounding effects, a multivariate Cox proportional-hazards regression model was used to calculate the independent HRs with 95%CI for OS and PFS for PRO-C3, C1M, C3M, C4M and VICM individually after adjusting for the above described covariates. For both univariate and multivariate analysis, baseline PRO-C3, C1M, C3M and C4M levels lower than the 75th percentile (Q1-Q3) were used as a reference to calculate the HRs for patients with baseline levels in the upper quartile (Q4). Baseline VICM levels in the lowest quartiles (Q1-Q2) were used as a reference to calculate the HRs for patients with baseline levels in the upper quartiles (Q3-Q4). LDH levels under 250 IU/L were used as a reference to calculate the HRs for patients with elevated LDH levels. Univariate analysis also included HRs for PRO-C3, C1M, C3M, C4M and VICM on a continuous scale. The OS for the patients in this study ranged from 40 days to 1258 days and the PFS ranged from 10 days to 1258 days from baseline. Kaplan-Meier (KM) survival curves were used to analyze PFS and OS in the same percentile groups as described for the Cox proportional-hazards regression models. KM survival curves were also used to analyze OS in patients with a low type III collagen degradation to formation (C3M/PRO-C3) ratio (Q1) compared to a high C3M/PRO-C3 ratio (Q2 + Q3 + Q4). A log-rank test was used to determine differences between KM curves. Statistical analysis was conducted using MedCalc (v16.8.4) and GraphPad Prism (v7.01) (GraphPad Software). A *p*-value of *p* < 0.05 was considered statistically significant.

## Results

### Patient characteristics

A total of 66 metastatic melanoma patients were included in this study and biomarkers were measured in these patients at baseline and in 52 patients 3 weeks after IPI treatment. According to an assessment of the clinical response by RECIST, 41 patients had PD, 14 patients had SD, 9 patients had PR and 2 patients had CR. Of the 66 patients, 43 patients were deceased within the follow-up period. Baseline patient characteristics are shown in Table 1.

**Table 1:** Patient characteristics

Age at baseline (median with range)	67 (35–83)
Gender (% females)	36/66 (55%)
Prior systemic therapy:	
None	36
IL-2	12
Temozolomide	6
Radiation therapy/IL-2	1
Radiation therapy	1
Temozolomide/Vemorafenib	1
RT/Temozolomide	1
IFN/IL-2	6
Vemorafenib	2
RECIST response	
PD	41
SD	14
PR	9
CR	2
Lactate dehydrogenase (LDH)	
> = 250 IU/L	12
< 250 IU/L	53

*PD* Progressive disease, *SD* Stable disease, *PR* Partial response, *CR* Complete response

### High pre-treatment PRO-C3, C1M, C3M and C4M levels are associated with progressive disease

**Fig. 1:**
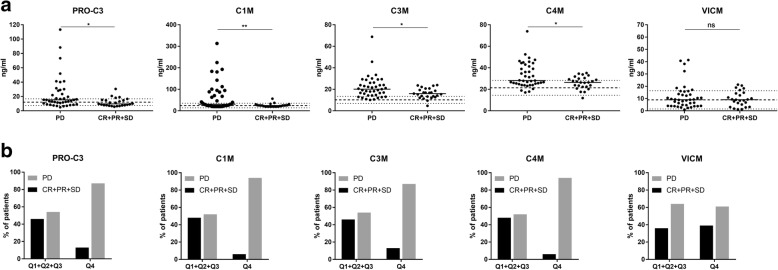
Relationship between PRO-C3, C1M, C3M, C4M and VICM levels at baseline and response. **a** Biomarker levels at baseline in serum from patients with progressive disease (PD) (*n* = 41) were compared to levels in patients with complete response (CR), partial response (PR) and stable disease (SD) (CR + PR + SD) (*n* = 25) with Mann-Whitney test. The black horizontal lines represent the median value of the patients. The reference limits for healthy individuals are illustrated with dotted lines (7.5–16.7 ng/ml, 14.0–35.6 ng/ml, 7–13.4 ng/ml, 14.5–28.3 ng/ml and 1.5–16.3 ng/ml for PRO-C3, C1M, C3M, C4M and VICM, respectively.) Asterisks indicate the following: *, *p < 0.05* and **, *p* < 0.01. **b** Serum levels were dichotomized by the 75th percentile for PRO-C3, C1M, C3M and C4M (Q4, *n* = 16) and by the median for VICM (Q3 + Q4, *n* = 33). Next, quantity of patients achieving disease control (CR + PR + SD) where compared to non-responders (PD) in each group

Serum concentrations of five different ECM biomarkers: PRO-C3, C1M, C3M, C4M and VICM, were measured at baseline. The majority of patients had biomarkers levels within the reference limits for healthy individuals, while a subgroup of patients had increased biomarker levels (Fig. 1a).

We investigated if the observed inter-patient variations in biomarker levels at baseline were associated with Disease Control Rate (DCR). When comparing the serum levels in patients progressing despite treatment (PD) to patients achieving disease control (CR + PR + SD), PRO-C3 (*p* = 0.011), C1M (*p* = 0.003), C3M (*p* = 0.013) and C4M levels (*p* = 0.027) at baseline were significantly elevated in patients with PD compared to the combined groups of patients with disease control (Fig. 1a). No significant difference was detected in VICM levels (*p* = 0.834). To investigate the association with DCR further, the biomarker levels were dichotomized by the 75th percentile cut-point (Q4) for PRO-C3, C1M, C3M and C4M and by the median for VICM and followed by a comparison of percentage of patients achieving disease control (CR + PR + SD) with non-responders (PD) in each group. Of the patients with PRO-C3, C1M, C3M and C4M levels in the upper quartile (Q4), only 13, 6, 13 and 6% obtained disease control (CR + PR + SD) compared to 46, 48, 46 and 48% in the group of patients with lower biomarker levels (Q1 + Q2 + Q3) (Fig. 1b). Of the patients with VICM levels in the upper quartiles (Q3 + Q4), 39% obtained disease control compared to 36% in the group with lower levels (Q1 + Q2).

ORs were subsequently used to calculate the odds of being diagnosed with PD compared to CR + PR + SD. High levels (Q4) of PRO-C3, C1M, C3M and C4M resulted in ORs for being in the PD group of 6 (95%CI = 1.2–28, *p* = 0.019), 14 (95%CI = 2.2–151.6, *p* = 0.003), 6 (95%CI = 1.2–28, p = 0.019) and 14 (95%CI = 2.2–151.6, p = 0.003), respectively. In line with this, PPVs for having PD were 0.88, 0.94, 0.88 and 0.94, respectively. No association between VICM and DCR was observed (OR = 0.88, 95%CI = 0.3–2.5, *p* > 0.999).

### PRO-C3, C4M and VICM are predictors of outcome

The individual ability of PRO-C3, C1M, C3M, C4M, VICM and clinical covariates at baseline to predict OS is shown in Table 2, which summarizes HRs with 95%CIs calculated from univariate Cox proportional-hazard models. When evaluated on a continuous scale, PRO-C3, C1M, C3M, C4M and LDH, but not VICM, were predictive of poor OS. When evaluated by the dichotomous cut-point (75th percentile cut-point, Q4), high pre-treatment levels of PRO-C3 (HR = 2.13, 95%CI = 1.12–4.04, *p* = 0.021) and C4M (HR = 2.43, 95%CI = 1.26–4.70, *p* = 0.008) were predictive of poor OS while a trend was seen for C1M (HR = 1.70, 95%CI = 0.85–3.38), *p* = 0.131) and C3M (HR = 1.60, 95%CI = 0.82–3.13, *p* = 0.167). In comparison, high VICM (Q3 + Q4) at baseline was predictive of a survival benefit (HR = 0.54, 95%CI = 0.29–0.99, *p* = 0.044). High LDH (> 250 IU/L) was the only covariate associated with poor OS (HR = 2.02, 95%CI = 0.99–4.12, *p* = 0.052). To determine the potential independent predictive value of PRO-C3, C1M, C3M, C4M and VICM for OS, multivariate Cox proportional-hazard models were used to calculate HRs for the biomarkers (dichotomized) adjusted for the covariates age, LDH and prior systemic treatment (Table 2). Of the collagen markers, only high PRO-C3 and C4M (Q4) were independently predicting poor OS (HR = 2.04, 95%CI = 1.00–4.16, *p* = 0.049 and HR = 2.18, 95%CI = 1.01–4.70, *p* = 0.046, for PRO-C3 and C4M, respectively). In contrast to PRO-C3 and C4M which predicted a poor outcome, high VICM (Q3 + Q4) was independently predictive of a survival benefit (HR = 0.49, 95%CI = 0.26–0.92, *p* = 0.026).

**Table 2:** Association between biomarkers at baseline, clinical covariates and overall survival for metastatic melanoma patients

Variable		HR	95%Cl	*p*-value
PRO-C3	Continuous	1.03	1.02–1.05	0.0004
	5.0–19.2 ng/ml, Q1-Q3	1.00	–	–
Univariate	19.6–113.3 ng/ml, Q4	2.13	1.12–4.04	0.021
	5.0–19.2 ng/ml, Q1-Q3*	1.00	–	–
Multivariate	19.6–113.3 ng/ml, Q4*	2.04	1.00–4.16	0.049
C1M	Continuous	1.01	1.00–1.01	0.005
	20–46.6 ng/ml, Q1-Q3	1.00	–	–
Univariate	56.7–313.1 ng/ml, Q4	1.70	0.85–3.38	0.131
	20–46.6 ng/ml, Q1-Q3*	1.00	–	–
Multivariate	56.7–313.1 ng/ml, Q4*	1.17	0.54–2.51	0.693
C3M	Continuous	1.03	1.00–1.07	0.037
	4.7–23.4, Q1-Q3	1.00	–	–
Univariate	23.6–68.8, Q4	1.60	0.82–3.13	0.167
	4.7–23.4, Q1-Q3*	1.00	–	–
Multivariate	23.6–68.8, Q4*	1.06	0.48–2.33	0.886
C4M	Continuous	1.04	1.01–1.08	0.026
	11.9–34.7 ng/ml, Q1-Q3	1.00	–	–
Univariate	35.1–73.9 ng/ml, Q4	2.43	1.26–4.70	0.008
	11.9–34.7 ng/ml, Q1-Q3*	1.00	–	–
Multivariate	35.1–73.9 ng/ml, Q4*	2.18	1.01–4.70	0.046
VICM	Continuous	0.99	0.95–1.02	0.413
	1.0–9.1 ng/ml, Q1-Q2	1.00	–	–
Univariate	9.1–41.3 ng/ml, Q3-Q4	0.54	0.29–0.99	0.044
	1.0–9.1 ng/ml, Q1-Q2*	1.00	–	–
Multivariate	9.1–41.3 ng/ml, Q3-Q4*	0.49	0.26–0.92	0.026
Age at baseline		1.02	0.99–1.04	0.254
LDH at sampling	Continuous	1.00	1.00–1.00	0.009
	(> = 250 IU/L)	2.02	0.99–4.12	0.052
Prior systemic therapy		1.32	0.73–2.40	0.363

Hazard ratios (HR) were calculated by univariate and multivariate analysis (indicated by stars). By the univariate analysis, biomarkers were analyzed on both a continuous scale and divided into quartiles with the lower quartiles (Q1-Q3) or (Q1-Q2) used as a reference to calculate the HR for patients in the upper quartiles (Q4) or (Q3-Q4). All covariates were analyzed on a continuous scale and LDH was furthermore analyzed on a binominal scale. By the multivariate analysis, the individual biomarkers were adjusted for the covariates age, LDH and prior systemic treatment. *LDH* Lactate dehydrogenase

The biomarker levels at baseline were also assessed for their prediction of PFS. In brief, similar results as for OS were observed, with the exception of C1M levels (Additional file 1: Table S1). Interestingly for C1M, although high C1M (Q4) levels were not significantly predictive of OS (Table 2), high C1M levels were predictive of PFS by the univariate analysis (HR = 2.13, 95%CI = 1.17–3.88, *p* = 0.013) and borderline significant with the multivariate analysis (HR = 1.84, 95%CI = 0.97–3.51, *p* = 0.064) (Additional file 1: Table S1).

### PRO-C3, C4M and VICM at baseline are associated with survival over time

**Fig. 2:**
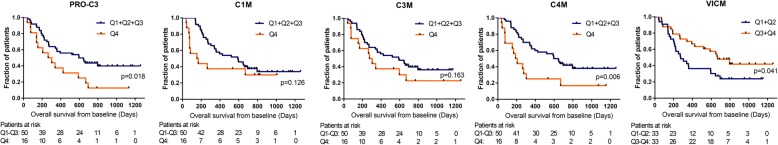
Kaplan-Meier analysis of overall survival in ipilimumab treated melanoma patients. Overall survival for patients with biomarker levels in the upper quartile (Q4) vs the lower quartiles (Q1 + Q2 + Q3) for PRO-C3, C1M, C3M and C4M, while for VICM it is the upper quartiles (Q3 + Q4) vs the lower quartiles (Q1 + Q2). A log-rank test was used to determine differences between the survival curves where a p-value of *p* < 0.05 was considered statistically significant

KM survival plots show the OS within the follow-up period according to baseline levels for the five analyzed biomarkers (Fig. 2). High baseline levels (Q4) of PRO-C3 (*p* = 0.018) and C4M (*p* = 0.006) were significantly associated with shorter OS while a trend was seen for C1M (*p* = 0.126) and C3M (*p* = 0.163). The median OS was 285, 161, 290 or 198 days in biomarker high patients versus 596, 592, 592 or 621 days in biomarker low patients for PRO-C3, C1M, C3M and C4M, respectively. In contrast, high levels of VICM (Q3 + Q4) were associated with longer OS (*p* = 0.041) with a median OS of 669 days versus 275 days in VICM low patients. When assessing PFS curves comparable findings were observed (Additional file 1: Figure S1). Interestingly, when evaluating OS after 1 year, high baseline levels of C1M (Q4) were significantly associated with poor OS (*p* = 0.016) (Additional file 1: Figure S2).

### High C3M/PRO-C3 ratio at baseline associates with increased overall survival

**Fig. 3:**
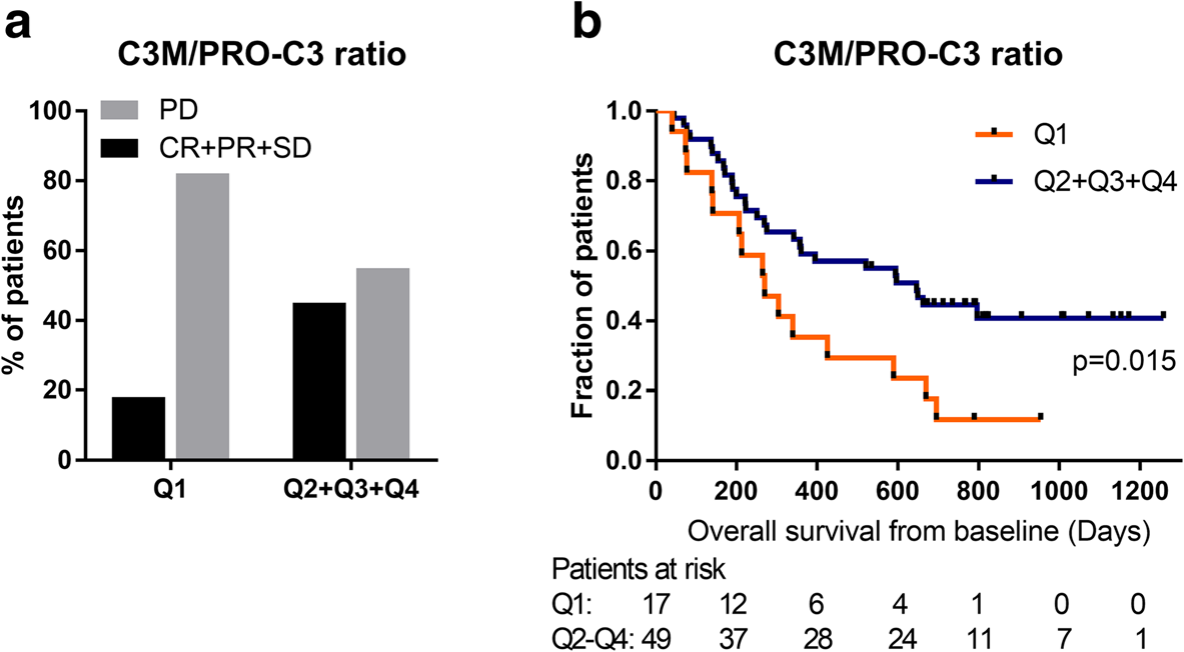
Relationship between C3M/PRO-C3 ratio at baseline and clinical outcome. C3M/PRO-C3 serum levels were dichotomized by the 25th percentile dividing the patients into a group with low C3M/PRO-C3 (Q1) and high C3M/PRO-C3 (Q2 + Q3 + Q4). **a** Quantity of patients achieving disease control (CR + PR + SD) compared to non-responders (PD) in each group. **b** Kaplan-Meier survival curve illustrate the overall survival for the two groups. A log-rank test was used to determine differences between the survival curves where a *p*-value of *p* < 0.05 was considered statistically significant

Next, we examined if the ratio of type III collagen degradation to formation (C3M/PRO-C3) at baseline could give additional information when looking on DCR and OS. We dichotomized the C3M/PRO-C3 serum levels by the 25th percentile (Q1 vs Q2 + Q3 + Q4) based on the observation that high PRO-C3 (Q4) was significantly associated with shorter OS compared to low PRO-C3 (Q1 + Q2 + Q3) suggesting that a low C3M/PRO-C3 ratio likewise was associated with decreased OS. Low levels of C3M/PRO-C3 (Q1) were able to differentiate PD from CR + PR + SD with an OR of 3.8 (95%CI = 1.0–13.5, *p* = 0.080) and a PPV of 0.82 (Fig. 3a). When looking at survival outcome, a high C3M/PRO-C3 ratio at baseline (Q2 + Q3 + Q4) was significantly associated with longer OS when assessed by a log-rank test (*p* = 0.015) (Fig. 3b) and the multivariate Cox proportional-hazard analysis (HR = 0.47, 95%CI = 0.24–0.95, *p* = 0.034). We assessed the correlation between the C3M/PRO-C3 ratio and PRO-C3 or C3M. It was demonstrated that a low level of PRO-C3 and an intermediate level of C3M drive a high C3M/PRO-C3 ratio (Additional file 1: Figure S3).

### Changes in C3M and VICM levels after 3 weeks of treatment are not associated with overall survival

**Fig. 4:**
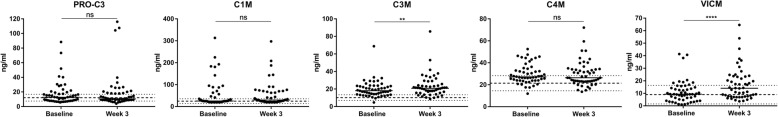
Biomarker levels in serum during treatment. PRO-C3, C1M, C3M, C4M and VICM levels in serum at baseline (*n* = 52) and 3 weeks after ipilimumab treatment (*n* = 52). The black horizontal lines represent the median value of the patients. Serum levels were compared using Wilcoxon matched-pairs rank test. Asterisks indicate the following: **, *p* < 0.01 and ****, *p* < 0.0001

Serum concentrations of PRO-C3, C1M, C3M, C4M and VICM at baseline and at week 3 are shown in Fig. 4 (Raw data are shown in Additional file 1: Table S2). When the biomarker levels were paired, neither PRO-C3 (*p* = 0.735), C1M (*p* = 0.811) nor C4M (*p* = 0.642) showed significant changes during 3 weeks of treatment whereas significantly elevated levels of C3M (*p* = 0.003) and VICM (*p* < 0.0001) were observed at the 3 week time-point.

Based on the observation of significantly elevated C3M and VICM levels during treatment, the OS was assessed according to percentage decrease or increase in biomarker levels from baseline to 3 weeks of treatment. The change (increase vs. decrease) in these biomarkers did not associate with OS: C3M (HR = 1.07, 95%CI = 0.52–2.18, *p* = 0.861) and VICM (HR = 2.17, 95%CI = 0.89–5.28, *p* = 0.089), albeit a trend was seen for VICM.

## Discussion

In the present study, we measured a panel of five ECM biomarkers in serum from patients with stage IV malignant melanoma treated with IPI. The main findings showed that high baseline levels (Q4) of the collagen biomarkers PRO-C3, C1M, C3M and C4M were associated with poor response (according to RECIST) to IPI. In addition, high PRO-C3 and C4M (Q4) were associated with shorter OS. In contrast, high VICM (Q3 + Q4) at baseline was linked to a survival benefit. Application of the ratio of type III collagen degradation to formation (C3M/PRO-C3) added additional information with the observation that a high C3M/PRO-C3 ratio (Q2 + Q3 + Q4) was associated with longer OS.

ICIs induce only durable responses in a minority of patients, and is associated with significant risk of inflammatory toxicity and high costs. Therefore, biomarkers for prediction of response and resistance represent unmet needs. Factors as mutational load [29], PD-L1 expression [30], cytokines [31], blood immune cells [24, 32] and TILs [33] have been identified to correlate with treatment response to ICIs. However, tumor heterogeneity, technical issues and the complexity of the immune response limit the applicability of these biomarkers. There is increasing interest in exploring the stromal matrix components for its role in regulating anti-tumor immune responses [4]. To our knowledge, this is the first study to show the biomarker potential of quantifying altered ECM remodeling in a liquid biopsy (serum) for predicting outcome to ICIs. The ECM biomarkers in this study are measured by technical robust ELISAs, a relative simple and inexpensive technology, which accurately measure the analytes of interest [17, 25–28]. Peripheral blood biomarkers have an advantage due to the ease of accessing blood versus tumor tissue. Furthermore, it is presumed that ECM fragments are more stable in the circulation than for instance cytokines, which depend on the immune status of the patient.

Elevated levels of collagen-derived fragments reflecting an imbalance in ECM have previously been linked to cancer progression [18, 20, 22]. C1M and C3M reflect interstitial matrix degradation, which may pave the way for tumor cell migration and progression. In this study, high baseline levels of C1M and C3M were associated with PD and a trend was seen for an association to poor OS. However, when adjusting for covariates as LDH, high C1M and C3M levels were not independently linked to poor OS. Elevated serum LDH indicates tissue damage, which triggers inflammation and degradation of the ECM, which may account for the association. High LDH levels are an indicator of poor response to IPI in stage IV melanoma patients [34, 35].

It has previously been shown that ICI treatment can lead to immune-related adverse events with clinical manifestations similar to rheumatic disease [36]. In this study, an increase in C3M levels after IPI treatment was demonstrated, which may be associated with increased inflammation. In line with this, it has been shown that C3M levels are higher in rheumatoid arthritis patients than in healthy controls [37]. This link between potential IPI-induced C3M levels and rheumatic arthritis should be investigated in further studies.

Excessive collagen deposition is important for tumor progression and has been linked to a blocking of the T-cell recruitment, which is important for efficient ICI therapy [38, 39]. In the present study, high levels of PRO-C3 (which measures true formation of type III collagen [17]) at baseline were associated with PD and poor OS, thereby suggesting that a dense fibrotic network blocks T-cell infiltration which would contribute to the resistance to anti-CTLA-4 treatment. In a recent study, the lack of response to anti-PD-L1 treatment was associated with transforming growth factor-β (TGF-β) signaling in fibroblasts, and this occurred particularly in patients with T-cell excluded tumors where T cells got trapped in the surrounding collagen [8]. TGF-β promotes production of ECM components and it was furthermore shown that the co-administration of TGF-β-blocking and anti-PD-L1 antibodies to an immune-excluded mouse model facilitated T-cell penetration into tumors and tumor regression [8]. This highlights the prominent role of ECM deposition in reducing anti-tumor immunity and furthermore the potential for combination therapies with stroma targeting agents.

The role of altered collagen degradation to formation for response to IPI was investigated in our study by looking at the C3M/PRO-C3 ratio at baseline. Interestingly, a high C3M/PRO-C3 ratio predicts good outcome, highlighting that the balance between ECM degradation to formation is important and indicating that the ratio/balance is clinically informative for outcome measures in malignant melanoma patients treated with IPI. This suggest that patients with less net fibrosis/collagen deposition (PRO-C3) are more likely to respond to ICI treatment. In support, Wang et al. showed that the C3M/PRO-C3 ratio provided true predictive value for response to PEGPH20, a stromal modifier, in a phase II trial of patients with metastatic pancreatic cancer [40].

The collagen biomarker C4M reflects basement membrane remodeling, which is associated with malignant progression and metastatic dissemination [41]. The association between high baseline levels of C4M and poor outcome supports that basement membrane remodeling contributes to PD. The collagen turnover biomarkers in this study have high PPVs for identifying patients with PD, which support their applicability for excluding a subpopulation of patients with poor chance of responding.

Several studies have indicated that collagen fragments have biological activity in which fragments of collagens binds to integrins or ITIM-bearing receptors resulting in pro- or anti-tumorigenic responses [14, 42]. LAIR-1 is an ITIM receptor expressed by PBMCs that binds collagens, which negatively regulate the immune response [43]. A mechanism that could maintain infiltrating immune cells in a suppressive pro-tumorigenic state. ECM bioactive fragments can also be chemotactic for myeloid cells, like neutrophils, which are associated with poor prognostic outcome and short OS in melanoma patients undergoing immunotherapy [44–46]. Protease-derived collagen fragments from type I and IV collagen have previously been shown to promote neutrophil chemotaxis suggesting that elevated C1M and C4M might contribute to an immunosuppressive tumor microenvironment, which leads to poor outcome [47].

We demonstrated that high VICM levels at baseline were associated with longer OS indicating a role of macrophages in response to IPI. In support of this, it has been shown that melanoma patients responding to IPI display higher frequencies of tumor-infiltrating macrophages at baseline compared with non-responding patients [48].

Although we discovered a change in C3M and VICM levels after 3 weeks of treatment, only a trend towards an association with OS was seen for VICM. It is possible that 3 weeks might not be enough time for inducing sufficient change in biomarker levels associated with a difference in outcome and further analyses are warranted.

The present study is limited by that half of the patients were initially treated with systemic therapy before receiving IPI. However, when we adjusted for prior systemic therapy in the multivariate analysis, high levels of PRO-C3 and C4M were independently predictive of poor OS while high levels of VICM were independently predictive for longer OS suggesting that prior systemic therapy does not affect the potential of these biomarkers.

This study was a retrospective analysis, which needs to be validated using larger patient cohorts and different types of cancer and ICI therapies. These neo-epitope biomarkers reflecting changes of the ECM can probably not be used to guide decisions about immunotherapy straight away but they have indeed potential for being promising biomarkers. Furthermore, these preliminary results can improve the scientific understanding of ECM as being a vital factor that influence response to ICIs.

## Conclusions

ICIs induce only durable responses in a subset of metastatic melanoma patients suggesting that the clinical response is indeed complex and multifactorial. Emerging evidence suggests that the ECM and proteolytic ECM remodeling products have a crucial role in regulating the cancer-immunity cycle. We show that stromal matrix components can be used as biomarkers in an ICI setting. High PRO-C3, C1M, C3M and C4M levels at baseline are associated with poor response to IPI while high VICM is associated with longer OS. This explorative study provides knowledge about ECM as a main player in anti-tumor immune responses and suggests that ECM-derived biomarkers have the potential of improving patient stratification for ICI treatment.

## Additional file

Additional file 1: **Table S1.** Association between biomarkers at baseline, clinical covariates and progression free survival for metastatic melanoma patients. **Figure S1.** Kaplan-Meier analysis of progression free survival in ipilimumab treated melanoma patients. **Figure S2.** Kaplan-Meier analysis of overall survival in ipilimumab treated melanoma patients. **Figure S3.** Correlation between the C3M/PRO-C3 ratio and levels of C3M or PRO-C3 in the individual melanoma patients. **Table S2.** Biomarker levels in serum at baseline and 3 weeks after ipilimumab treatment. (DOCX 238 kb)

## Abbreviations

C1M: MMP-degraded type I collagen
C3M: MMP-degraded type III collagen
C3M/PRO-C3: Type III collagen degradation to formation
C4M: MMP-degraded type IV collagen
CR: Complete response
CTLA-4: Cytotoxic T lymphocyte antigen 4
DCR: Disease control rate
ECM: Extracellular matrix
ELISA: Enzyme-linked immunosorbent assay
HR: Hazard ratio
ICI: Immune checkpoint inhibitor
IPI: Ipilimumab
KM: Kaplan-Meier
LDH: Lactate dehydrogenase
MMP: Matrix metalloproteinase
OR: Odds ratio
OS: Overall survival
PD: Progressive disease
PD-1: Programmed cell death protein 1
PD-L1: PD1 ligand 1
PFS: Progression free survival
PPV: Positive predictive value
PR: Partial response
PRO-C3: Type III collagen formation
RECIST: Response Evaluation Criteria in Solid Tumors
SD: Stable disease
TGF-β: Transforming growth factor-β
TIL: Tumor infiltrating lymphocyte
VICM: Citrullinated and MMP-degraded vimentin
