# Supplementary material for: Non-invasive biomarkers derived from the extracellular matrix associate with response to immune checkpoint blockade (anti-CTLA-4) in metastatic melanoma patients
Source: J Immunother Cancer. 2018 Dec 19;6:152. doi: 10.1186/s40425-018-0474-z (PMC6300009; doi:10.1186/s40425-018-0474-z)
Supplement: Supplementary file 1 — Table S1. Association between biomarkers at baseline, clinical covariates and progression free survival for metastatic melanoma patients. Figure S1. Kaplan-Meier analysis of progression free survival in ipilimumab treated melanoma patients. Figure S2. Kaplan-Meier analysis of overall survival in ipilimumab treated melanoma patients. Figure S3. Correlation between the C3M/PRO-C3 ratio and levels of C3M or PRO-C3 in the individual melanoma patients. Table S2. Biomarker levels in serum at baseline and 3 weeks after ipilimumab treatment. (DOCX 238 kb) [file 40425_2018_474_MOESM1_ESM.docx]

Additional file 1 for

**Non-invasive biomarkers derived from the extracellular matrix associate with response to immune checkpoint blockade (anti-CTLA-4) in metastatic melanoma patients**

Christina Jensen^1,2^, Daniel Hargbøl Madsen^3^, Morten Hansen^3^, Henrik Schmidt^4^, Inge Marie Svane^3^, Morten Asser Karsdal^1^, Nicholas Willumsen^1^

^1^Biomarkers & Research, Nordic Bioscience, Herlev Hovedgade 205-207, 2730 Herlev, Denmark
^2^Biotech Research & Innovation Centre (BRIC), University of Copenhagen, Ole Maaløes Vej 5, 2200 Copenhagen N, Denmark
^3^Center for Cancer Immune Therapy, Department of Haematology and Department of Oncology, Herlev Hospital, University of Copenhagen, Herlev Ringvej 75, 2730 Herlev, Denmark
^4^Department of Oncology, Aarhus University Hospital, Nørrebrogade 44, 8000 Aarhus C, Denmark

This file includes:

Supplementary Table 1. Association between biomarkers at baseline, clinical covariates and progression free survival for metastatic melanoma patients

Supplementary Figure 1. Kaplan-Meier analysis of progression free survival in ipilimumab treated melanoma patients.

Supplementary Figure 2. Kaplan-Meier analysis of overall survival in ipilimumab treated melanoma patients.

Supplementary Figure 3. Correlation between the C3M/PRO-C3 ratio and levels of C3M or PRO-C3 in the individual melanoma patients.

Supplementary Table 2. Biomarker levels in serum at baseline and three weeks after ipilimumab treatment

| **Supplementary Table 1** Association between biomarkers at baseline, clinical covariates and progression free survival for metastatic melanoma patients | | | | |
| --- | --- | --- | --- | --- |
| **Variable** |  | **HR** | **95%Cl** | **p-value** |
| **PRO-C3**  Univariate  Multivariate | Continuous 5.0-19.2 ng/ml, Q1-Q3 19.6-113.3 ng/ml, Q4  5.0-19.2 ng/ml, Q1-Q3* 19.6-113.3 ng/ml, Q4* | 1.03  1.00  1.90  1.00  2.04 | 1.01-1.04  -  1.06-3.40  -  1.08-3.87 | 0.001  -  0.030  -  0.028 |
| **C1M**  Univariate  Multivariate | Continuous 20-46.6 ng/ml, Q1-Q3 56.7-313.1 ng/ml, Q4 20-46.6 ng/ml, Q1-Q3* 56.7-313.1 ng/ml, Q4* | 1.01  1.00  2.13 1.00 1.84 | 1.00-1.01 - 1.17-3.88 - 0.97-3.51 | 0.002 - 0.013 - 0.064 |
| **C3M**  Univariate  Multivariate | Continuous 4.7-23.4, Q1-Q3 23.6-68.8, Q4  4.7-23.4, Q1-Q3* 23.6-68.8, Q4* | 1.03  1.00  1.35  1.00  1.04 | 1.00-1.06 - 0.75-2.43  -  0.51-2.10 | 0.025 - 0.321  -  0.924 |
| **C4M**  Univariate  Multivariate | Continuous 11.9-34.7 ng/ml, Q1-Q3 35.1-73.9 ng/ml, Q4  11.9-34.7 ng/ml, Q1-Q3* 35.1-73.9 ng/ml, Q4* | 1.03 1.00 1.93 1.00 2.10 | 1.00-1.07  -  1.08-3.45 - 1.09-4.05 | 0.032 -  0.027 - 0.028 |
| **VICM**  Univariate  Multivariate | Continuous 1.0-9.1 ng/ml, Q1-Q2 9.1-41.3 ng/ml, Q3-Q4 1.0-9.1 ng/ml, Q1-Q2* 9.1-41.3 ng/ml, Q3-Q4* | 1.00 1.00 0.72 1.00 0.70 | 0.97-1.03 - 0.44-1.20 - 0.40-1.19 | 0.998 - 0.204 - 0.188 |
| **Age at baseline** |  | 1.00 | 0.98-1.02 | 0.767 |
| **LDH at sampling** | Continuous (>=250 IU/L) | 1.00 2.29 | 1.00-1.00  1.19-4.41 | 0.003 0.013 |
| **Prior systemic therapy** |  | 0.89 | 0.53-1.48 | 0.646 |
| Hazard ratios (HR) were calculated by univariate and multivariate analysis (indicated by stars). By the univariate analysis, biomarkers were analyzed on both a continuous scale and divided into quartiles with the lower quartiles (Q1-Q3) or (Q1-Q2) used as a reference to calculate the HR for patients in the upper quartiles (Q4) or (Q3-Q4). All covariates were analyzed on a continuous scale and LDH was furthermore analyzed on a binominal scale. By the multivariate analysis, the individual biomarkers were adjusted for the covariates age, LDH and prior systemic treatment. LDH=Lactate dehydrogenase. | | | | |


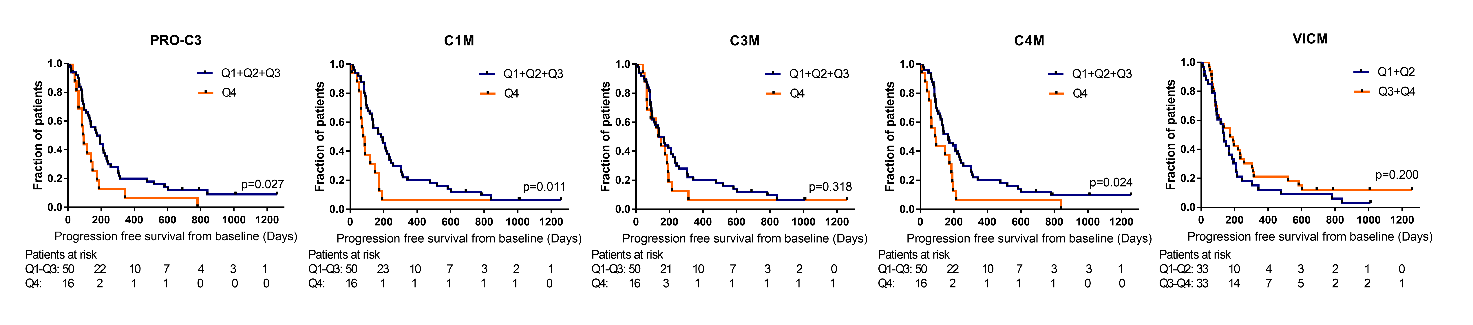


**Supplementary Figure 1** Kaplan-Meier analysis of progression free survival in ipilimumab treated melanoma patients. Progression free survival for patients with biomarker levels in the upper quartile (Q4) vs the lower quartiles (Q1+Q2+Q3) for PRO-C3, C1M, C3M and C4M, while for VICM it is the upper quartiles (Q3+Q4) vs the lower quartiles (Q1+Q2). A log-rank test was used to determine differences between the survival curves where a p-value of p<0.05 was considered statistically significant

**
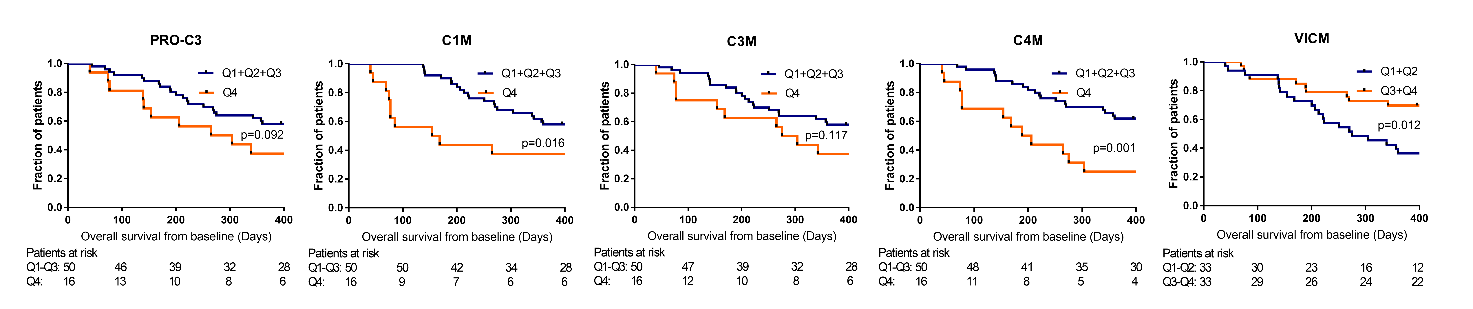
**

**Supplementary Figure 2** Kaplan-Meier analysis of overall survival in ipilimumab treated melanoma patients.

Overall survival for patients with biomarker levels in the upper quartile (Q4) vs the lower quartiles (Q1+Q2+Q3) for PRO-C3, C1M, C3M and C4M, while for VICM it is the upper quartiles (Q3+Q4) vs the lower quartiles (Q1+Q2). A log-rank test was used to determine differences between the survival curves where a p-value of p<0.05 was considered statistically significant


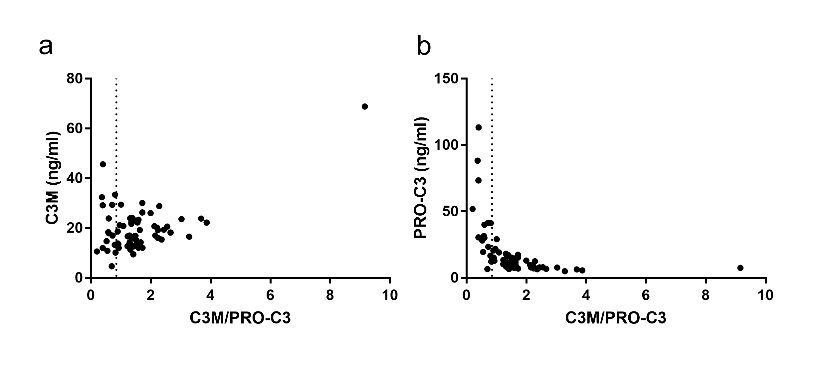


**Supplementary Figure 3** Correlation between the C3M/PRO-C3 ratio and levels of C3M or PRO-C3 in the individual melanoma patients. The correlation of the ratio of C3M/PRO-C3 and the biomarker levels of C3M (a) or PRO-C3 (b) in serum at baseline. The dotted vertical line represents the 25^th^ percentile cut-point for C3M/PRO-C3 (cut-point=0.85).

| **Supplementary Table 2** Biomarker levels in serum at baseline and three weeks after ipilimumab treatment | | | | | | | | | | |
| --- | --- | --- | --- | --- | --- | --- | --- | --- | --- | --- |
| **Patient** | **PRO-C3 Baseline** | **PRO-C3 Week 3** | **C1M Baseline** | **C1M Week 3** | **C3M Baseline** | **C3M Week 3** | **C4M Baseline** | **C4M Week 3** | **VICM Baseline** | **VICM Week 3** |
| 1 | 18.208 |  | 20.000 |  | 23.936 |  | 33.836 |  | 10.884 |  |
| 2 | 13.186 |  | 37.162 |  | 17.044 |  | 27.240 |  | 6.820 |  |
| 3 | 23.446 | 21.458 | 20.000 | 33.840 | 17.060 | 15.660 | 27.716 | 25.172 | 7.824 | 13.908 |
| 4 | 14.702 | 10.230 | 22.148 | 29.222 | 23.376 | 36.136 | 35.136 | 34.380 | 7.652 | 7.680 |
| 5 | 20.722 | 27.658 | 20.000 | 25.234 | 18.624 | 18.044 | 28.812 | 23.888 | 1.756 | 8.904 |
| 6 | 14.744 | 18.702 | 56.660 | 20.454 | 13.560 | 20.508 | 30.520 | 23.568 | 18.276 | 23.724 |
| 7 | 15.300 | 11.896 | 24.920 | 23.118 | 26.296 | 23.516 | 44.400 | 33.240 | 10.404 | 9.224 |
| 8 | 13.012 | 14.712 | 95.258 | 29.584 | 26.008 | 24.184 | 43.060 | 32.016 | 13.692 | 18.028 |
| 9 | 21.920 | 17.772 | 24.510 | 20.000 | 21.152 | 18.368 | 26.684 | 23.420 | 4.628 | 7.472 |
| 10 | 73.392 | 116.000 | 87.118 | 70.988 | 29.192 | 29.476 | 44.376 | 39.756 | 4.044 | 8.180 |
| 11 | 88.352 | 104.270 | 192.826 | 76.930 | 32.464 | 35.496 | 47.444 | 51.028 | 13.224 | 27.572 |
| 12 | 6.464 | 6.146 | 24.418 | 20.000 | 23.836 | 13.944 | 36.328 | 20.208 | 3.380 | 8.524 |
| 13 | 15.336 | 13.210 | 41.390 | 90.726 | 23.052 | 28.772 | 45.032 | 50.968 | 16.044 | 19.144 |
| 14 | 19.620 | 25.532 | 20.000 | 71.274 | 10.892 | 19.080 | 23.964 | 23.472 | 38.168 | 36.224 |
| 15 | 7.932 | 8.984 | 25.506 | 142.592 | 19.364 | 21.656 | 26.128 | 33.980 | 20.564 | 53.868 |
| 16 | 8.582 | 6.396 | 313.062 | 297.188 | 19.240 | 17.844 | 26.236 | 33.628 | 9.640 | 64.544 |
| 17 | 17.244 | 13.630 | 94.720 | 73.818 | 24.008 | 21.956 | 31.316 | 43.424 | 12.348 | 17.124 |
| 18 | 11.502 | 10.442 | 20.000 | 24.196 | 16.756 | 10.360 | 26.452 | 30.368 | 10.096 | 10.956 |
| 19 | 5.040 |  | 26.118 |  | 16.568 |  | 28.096 |  | 6.248 |  |
| 20 | 6.684 | 4.050 | 22.634 | 26.974 | 9.488 | 12.860 | 29.960 | 34.948 | 5.136 | 6.076 |
| 21 | 113.278 |  | 112.450 |  | 45.616 |  | 73.896 |  | 2.916 |  |
| 22 | 41.270 | 107.568 | 183.422 | 62.794 | 33.400 | 52.956 | 45.624 | 72.000 | 18.584 | 6.896 |
| 23 | 28.192 | 26.622 | 22.880 | 30.736 | 14.744 | 21.500 | 23.944 | 32.688 | 3.772 | 21.736 |
| 24 | 51.990 | 32.810 | 24.800 | 30.916 | 10.640 | 21.520 | 20.400 | 24.324 | 2.400 | 4.544 |
| 25 | 17.488 | 13.018 | 35.380 | 25.198 | 30.084 | 15.528 | 26.724 | 24.408 | 9.076 | 6.960 |
| 26 | 9.094 | 9.896 | 20.000 | 20.902 | 20.144 | 22.620 | 25.692 | 19.920 | 9.304 | 17.340 |
| 27 | 7.894 | 10.330 | 25.250 | 20.000 | 17.000 | 17.816 | 26.360 | 23.084 | 9.256 | 11.256 |
| 28 | 15.354 | 25.726 | 24.998 | 37.622 | 13.848 | 15.468 | 23.452 | 23.128 | 3.204 | 7.712 |
| 29 | 6.532 | 10.540 | 32.708 | 63.390 | 15.440 | 16.860 | 23.848 | 21.544 | 9.356 | 17.284 |
| 30 | 8.076 | 8.420 | 26.756 | 24.468 | 20.604 | 20.224 | 32.336 | 22.772 | 6.332 | 7.440 |
| 31 | 7.144 | 7.192 | 31.640 | 20.000 | 16.036 | 41.864 | 31.876 | 34.016 | 8.344 | 4.952 |
| 32 | 12.934 | 20.140 | 20.000 | 20.000 | 12.052 | 8.672 | 18.928 | 15.148 | 4.056 | 7.032 |
| 33 | 15.538 |  | 20.000 |  | 13.120 |  | 23.104 |  | 3.800 |  |
| 34 | 29.182 | 39.676 | 224.486 | 208.236 | 29.424 | 37.812 | 41.268 | 59.472 | 6.616 | 34.376 |
| 35 | 41.268 |  | 39.744 |  | 29.388 |  | 49.292 |  | 3.944 |  |
| 36 | 7.006 | 9.174 | 46.598 | 38.760 | 12.096 | 18.112 | 27.876 | 23.848 | 15.736 | 19.688 |
| 37 | 12.618 | 13.078 | 88.440 | 44.124 | 28.828 | 33.544 | 46.280 | 31.132 | 16.076 | 14.340 |
| 38 | 9.210 | 10.308 | 20.000 | 22.736 | 14.244 | 21.100 | 22.300 | 26.228 | 12.612 | 10.252 |
| 39 | 10.992 | 10.604 | 63.260 | 20.000 | 14.268 | 11.428 | 26.900 | 16.564 | 12.928 | 9.000 |
| 40 | 13.594 | 17.918 | 20.730 | 20.000 | 16.724 | 23.060 | 23.816 | 30.672 | 7.520 | 5.664 |
| 41 | 9.196 |  | 31.296 |  | 12.952 |  | 17.896 |  | 8.880 |  |
| 42 | 5.742 | 5.842 | 20.000 | 41.196 | 22.216 | 23.192 | 34.540 | 36.536 | 2.840 | 16.768 |
| 43 | 30.550 | 9.234 | 21.862 | 20.000 | 12.052 | 11.024 | 20.216 | 18.816 | 3.048 | 10.476 |
| 44 | 16.030 |  | 67.658 |  | 21.712 |  | 34.728 |  | 17.136 |  |
| 45 | 16.524 | 18.134 | 142.424 | 144.702 | 22.360 | 32.004 | 32.140 | 40.120 | 19.480 | 23.380 |
| 46 | 8.880 | 9.286 | 30.248 | 23.904 | 12.728 | 21.380 | 27.840 | 23.120 | 14.216 | 14.532 |
| 47 | 9.756 | 8.730 | 24.758 | 26.392 | 20.756 | 29.144 | 29.288 | 31.180 | 18.952 | 24.932 |
| 48 | 12.346 |  | 24.006 |  | 10.164 |  | 28.356 |  | 2.776 |  |
| 49 | 8.590 |  | 20.000 |  | 14.312 |  | 17.092 |  | 4.872 |  |
| 50 | 31.520 |  | 29.076 |  | 18.352 |  | 36.092 |  | 8.508 |  |
| 51 | 10.124 | 7.260 | 24.796 | 26.960 | 15.268 | 13.512 | 17.620 | 19.836 | 16.896 | 14.144 |
| 52 | 7.586 | 9.460 | 20.000 | 187.444 | 12.092 | 23.904 | 17.516 | 44.316 | 6.396 | 37.576 |
| 53 | 19.166 | 6.396 | 20.000 | 90.042 | 20.836 | 19.968 | 27.376 | 27.556 | 8.480 | 45.576 |
| 54 | 30.230 | 11.060 | 20.440 | 23.310 | 17.996 | 34.880 | 25.504 | 31.516 | 10.524 | 12.888 |
| 55 | 11.726 |  | 20.000 |  | 19.208 |  | 25.980 |  | 32.300 |  |
| 56 | 7.802 | 10.296 | 26.036 | 33.628 | 23.644 | 21.392 | 32.968 | 27.536 | 14.772 | 31.056 |
| 57 | 8.598 |  | 26.430 |  | 11.360 |  | 23.700 |  | 21.304 |  |
| 58 | 11.560 | 9.462 | 25.692 | 30.060 | 15.988 | 18.124 | 25.272 | 22.896 | 40.676 | 20.296 |
| 59 | 11.298 | 9.462 | 25.478 | 21.756 | 16.876 | 16.968 | 22.448 | 18.036 | 9.084 | 6.908 |
| 60 | 7.514 | 9.012 | 70.830 | 76.448 | 68.816 | 85.592 | 52.472 | 40.876 | 1.030 | 3.924 |
| 61 | 40.118 | 18.344 | 180.994 | 20.000 | 23.868 | 20.460 | 39.088 | 23.132 | 41.316 | 24.004 |
| 62 | 14.304 |  | 100.896 |  | 22.320 |  | 38.940 |  | 3.808 |  |
| 63 | 6.718 | 7.624 | 20.000 | 30.696 | 4.684 | 10.012 | 11.900 | 13.660 | 1.308 | 3.644 |
| 64 | 16.648 | 20.634 | 20.000 | 20.000 | 13.276 | 12.504 | 20.788 | 14.988 | 1.252 | 6.104 |
| 65 | 10.412 | 9.428 | 30.718 | 69.762 | 12.748 | 13.332 | 23.764 | 26.388 | 9.328 | 23.112 |
| 66 | 6.858 | 7.720 | 30.578 | 67.606 | 18.256 | 25.684 | 24.852 | 26.356 | 10.560 | 24.712 |
